# Restoration of a Canopy-Forming Alga Based on Recruitment Enhancement: Methods and Long-Term Success Assessment

**DOI:** 10.3389/fpls.2018.01832

**Published:** 2018-12-10

**Authors:** Jana Verdura, Marta Sales, Enric Ballesteros, Maria Elena Cefalì, Emma Cebrian

**Affiliations:** ^1^Facultat de Ciències, Institut d’Ecologia Aquàtica, Universitat de Girona, Girona, Spain; ^2^Estació d’Investigació Jaume Ferrer, Instituto Español de Oceanografía (IEO), Mahón, Spain; ^3^Centre d’Estudis Avançats de Blanes, CSIC, Blanes, Spain

**Keywords:** conservation, cost-effective restoration, *Cystoseira*, Fucales, human impacts, marine forests, recruitment enhancement, seaweed restoration

## Abstract

Marine forests dominated by macroalgae have experienced noticeable regression along some temperate and subpolar rocky shores. Along continuously disturbed shores, where natural recovery is extremely difficult, these forests are often permanently replaced by less structured assemblages. Thus, implementation of an active restoration plan emerges as an option to ensure their conservation. To date, active transplantation of individuals from natural and healthy populations has been proposed as a prime vehicle for restoring habitat-forming species. However, given the threatened and critical conservation status of many populations, less invasive techniques are required. Some authors have experimentally explored the applicability of several non-destructive techniques based on recruitment enhancement for macroalgae restoration; however, these techniques have not been effectively applied to restore forest-forming fucoids. Here, for the first time, we successfully restored four populations of *Cystoseira barbata* (i.e., they established self-maintaining populations of roughly 25 m^2^) in areas from which they had completely disappeared at least 50 years ago using recruitment-enhancement techniques. We compared the feasibility and costs of active macroalgal restoration by means of *in situ* (wild-collected zygotes and recruits) and *ex situ* (provisioning of lab-cultured recruits) techniques. Mid/long-term monitoring of the restored and reference populations allowed us to define the best indicators of success for the different restoration phases. After 6 years, the densities and size structure distributions of the restored populations were similar and comparable to those of the natural reference populations. However, the costs of the *in situ* recruitment technique were considerably lower than those of the *ex situ* technique. The restoration method, monitoring and success indicators proposed here may have applicability for other macroalgal species, especially those that produce rapidly sinking zygotes. Recruitment enhancement should become an essential tool for preserving *Cystoseira* forests and their associated biodiversity.

## Introduction

Canopy-forming brown macroalgae, such as kelps (Laminariales) and fucoids (Fucales), are habitat-forming species in the intertidal and subtidal zones of most temperate and subpolar regions (Steneck et al., 2002; Schiel and Foster, 2006). These macroalgae create structurally complex communities that have several similarities with terrestrial forests (Dayton et al., 1984, 1992; Ballesteros et al., 2009; Reed and Foster, 2012; Gianni et al., 2013). In addition to playing a crucial role in coastal primary production and nutrient cycling, these marine forests increase the three-dimensional complexity and spatial heterogeneity of rocky bottoms, providing food, shelter, nurseries and habitat for many other species (e.g., fish, invertebrates and other algae); thus, they host high biodiversity (Mann, 1973; Seed and O’Connor, 1981; Dayton, 1985; Graham, 2004; Schiel and Foster, 2006).

Compared to many other structurally complex ecosystems around the world, marine forests are suffering from a small global decline on average, despite large regional variation in both the direction and magnitude of the changes, meaning that while global declines are small on average, local-scale declines can be severe (Krumhansl et al., 2016). In many areas, the cumulative impacts of different human pressures, such as habitat destruction, pollution, overgrazing, invasive species and ocean warming, have largely disturbed canopy-forming macroalgae in recent decades (Steneck et al., 2002; Thibaut et al., 2005; Airoldi and Beck, 2007; Connell et al., 2008; Ling et al., 2009; Vergés et al., 2014, 2016; Wernberg et al., 2016). As a result, vast underwater marine forests have gone missing from many coastal areas and are being replaced by simpler and less productive communities dominated by opportunistic taxa (such as turfs or barrens) (Benedetti-Cecchi et al., 2001; Thibaut et al., 2005; Connell et al., 2008; Mangialajo et al., 2008; Ling et al., 2009; Smale and Wernberg, 2013; Vergés et al., 2014; Valdazo et al., 2017). Although some giant kelp populations have been shown to recover quickly from local- to large-scale disturbances (Dayton et al., 1992; Edwards, 2004), this is not always the case for other giant kelp populations, not for other kelps (e.g., Dayton, 1973) or fucoids (Coleman et al., 2008; Sales et al., 2011; Smale and Wernberg, 2013). The low dispersal abilities of zygotes and/or spores have been blamed for the lack of fucoid population recovery (Kendrick and Walker, 1991; Chapman, 1995; Dudgeon and Petraitis, 2001). In these cases, and when populations have become extinct, natural recovery is almost impossible, and active restoration emerges as the only tool to recover these missing forests (Stekoll and Deysher, 1996; Terawaki et al., 2003; Falace et al., 2006; Susini et al., 2007; Sales et al., 2011; Campbell et al., 2014).

The Mediterranean Sea, a marine biodiversity hotspot, has experienced large alterations in its ecosystems (Coll et al., 2010; Lotze et al., 2011). Marine forests dominated by species of the genus *Cystoseira* (Fucales) are widespread on well-preserved Mediterranean rocky bottoms (Giaccone, 1973; Ballesteros, 1988, 1990a,b; Ballesteros et al., 1998, 2009; Zabala and Ballesteros, 1989; Sales et al., 2012). Despite not reaching the size of kelp or some other fucoids, *Cystoseira* species produce a dense canopy (rarely > 1 m high) creating a “forest-like” assemblage, with species growing in the understory that are not found without their presence. This is the reason we speak about *Cystoseira* forests.

Some *Cystoseira* forests have severely declined in recent decades (Cormaci and Furnari, 1999; Thibaut et al., 2005; Serio et al., 2006; Blanfuné et al., 2016). Since zygotes of *Cystoseira* species are very large (around 100–120 μm) and exhibit low dispersal abilities (Guern, 1962; Clayton, 1992), transplantation techniques have been used as a tool for environmental mitigation (Falace et al., 2006; Susini et al., 2007; Perkol-Finkel et al., 2012; Robvieux, 2013).

However, since most *Cystoseira* species are considered threatened or endangered by the Barcelona Convention (Annex II) (United Nations Environment Programme/Mediterranean Action Plan [UNEP/MAP], 2013), individual transplants from remaining populations are undesirable, and therefore, less invasive restoration actions are required (see Gianni et al., 2013 for a review). As a result, new recruits of certain fucoid species have been artificially obtained and monitored for one year (Stekoll and Deysher, 1996; Terawaki et al., 2003; Yatsuya, 2010; Yu et al., 2012; Falace et al., 2018), introducing the possibility of recruitment enhancement as a new strategy for restoring *Cystoseira* populations.

In this context, the general objective of this study is to provide and experimentally test non-destructive restoration methods that can lead to the establishment of self-sustaining *Cystoseira* populations and to describe the proper success indicators for the different restoration stages. Specifically, we describe two techniques using *in situ* and *ex situ* recruitment enhancement aimed at restoring populations of *C. barbata*, and the success of each is assessed by comparing restored and reference populations over six years. Moreover, because the success and broad-scale application of a restoration technique also depends on its cost feasibility, we also describe this key piece of information.

## Materials and Methods

### Species and Study Site

This study focuses on the species *Cystoseira barbata* (Stackhouse) C. Agardh, which typically develops in shallow and sheltered environments (Sales and Ballesteros, 2009) across the Mediterranean Sea. The reduction in its range is strongly correlated with human development (Thibaut et al., 2005, 2015; Bologa and Sava, 2006), and the species is classified as threatened under the Barcelona Convention (United Nations Environment Programme/Mediterranean Action Plan [UNEP/MAP], 2013). These features make *C. barbata* a perfect target species for restoration in places from which it has disappeared.

**FIGURE 1:**
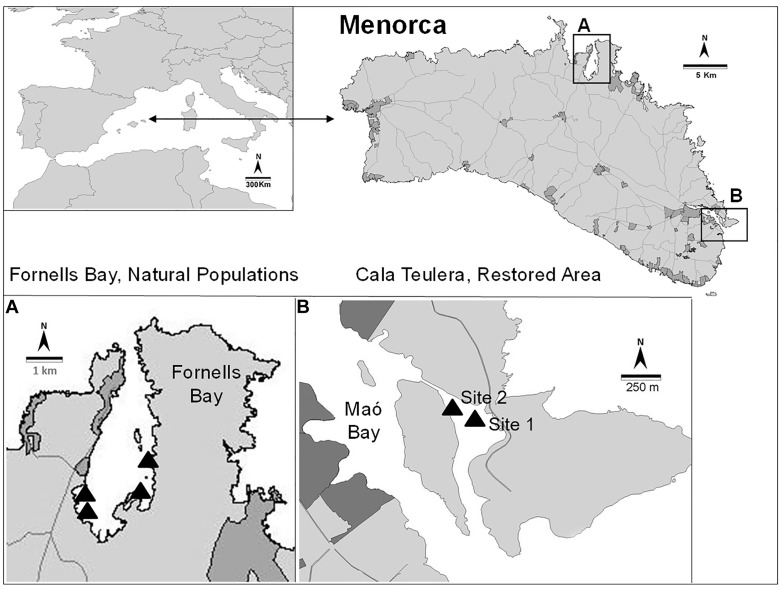
Location of the reference populations **(A)** and the restored area **(B)**.

This study was conducted in Menorca (Balearic Islands, NW Mediterranean), which has been a UNESCO Biosphere Reserve since 1993. Most coastal areas in Menorca are well preserved and have limited urbanization. The coastal water quality is high, so the extent and cover of habitats dominated by *Cystoseira* species is outstanding (Sales and Ballesteros, 2009). *Cystoseira barbata* naturally makes small patches (usually around a few square meters) in very sheltered and shallow environments. This species is extremely rare not only in Menorca but also in other Mediterranean areas (Gómez-Garreta et al., 2002) because there are very few places matching its environmental requirements, with the exception of the northern Adriatic Sea. *Cystoseira barbata* is present in Fornells Bay (Menorca), one of the few places where the environmental conditions are suitable for its development. However, this species was reported from Cala Teulera (39°52′40.64″ N, 4°18′22.03′ E; Bay of Maó, Figure 1) in the XVIII century (Rodríguez-Femenías, 1888), but it disappeared from this area due to direct dumping of urban and industrial sewage into the bay during the 1970s, leading to impaired water quality. A sewage outfall was built in 1980, and waste waters were diverted into the open sea (Hoyo, 1981). However, no recovery of the *C. barbata* populations was detected during the next 30 years (Sales et al., 2011). Nevertheless, Cala Teulera still shelters a reduced meadow of the seagrass *Cymodocea nodosa* and some stands of *Cystoseira compressa* var. *pustulata* and *Cystoseira foeniculacea* f. *tenuiramosa*. In contrast, Fornells Bay (40°2′10.12” N, 4°7′43.24′ E; Figure 1) continues to be characterized by low human influence and extensive sheltered seagrass meadows (e.g., *Posidonia oceanica, C. nodosa, Zostera noltii*) (Delgado et al., 1997) and healthy *Cystoseira* spp. forests, including the only preserved *C. barbata* populations from Menorca (Sales and Ballesteros, 2009). For this reason, the stands in Fornells Bay were selected as donor populations to restore two different sites in Cala Teulera (Figure 1).

### Applied Restoration Techniques

**FIGURE 2:**
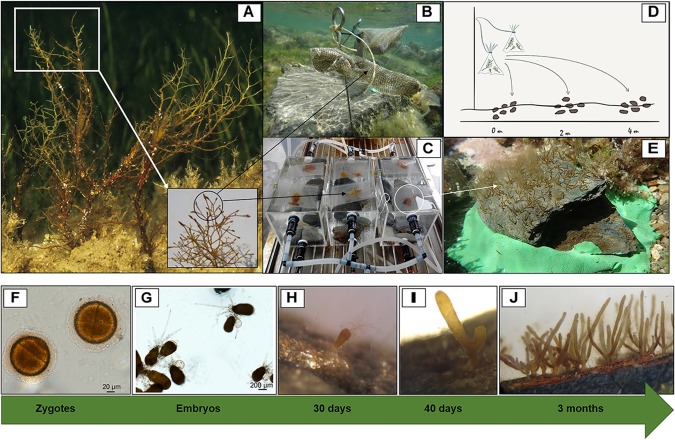
Experimental setup and zygote development into recruits. **(A)** Fertile thalli and branchlets from natural populations, **(B)** dispersal bags placed *in situ*, **(C)** dispersal bags placed in culture tanks (*ex situ*), **(D)** dispersion range capacity under *in situ* recruitment, and **(E)** placement of *ex situ* recruits in the area to be restored. Zygote and embryo development into recruits from *ex situ* cultures **(F–J).** **(F)** Zygotes (1 day), **(G)** embryos adhered to the substrate by rhizoids (1 week), **(H)** embryos developing into recruits (1 month, 200–400 μm), **(I)** first branching of the recruit (1.5 months, 400–600 μm), and **(J)** fully developed recruits (3 months, 5–15 mm).

Two different restoration techniques involving *in situ* and *ex situ* recruitment enhancement were experimentally tested to promote *C. barbata* recovery. Both techniques are considered non-destructive since they only rely on harvesting a small proportion (< 5%) of reproductive fertile branchlets from wild individuals. Both donor and restored sites were situated between depths of 0.2 m and 1 m. *In situ* recruitment consisted of collecting fertile apical branchlets (March 2011) from the donor populations (Fornells Bay) that were then transported to the restoration sites and placed in dispersal bags that were 8 cm wide and 10 cm long (Figures 2A,B) and made of 36% fiberglass and 64% PVC with a mesh size of 1.20 × 1.28 mm.

Bags were tied to a pick and directly fixed at a vertical distance of 0.25 cm from the bottom using a hammer (Figure 2B). Eight bags (two for each pick) containing approximately twenty fertile receptacles each were placed at each of the two selected restoration sites at distances of 2–3 m from each other. At both sites, six natural flat schist stones with similar surface areas (approximately 0.04 m^2^) were collected, cleaned of organisms and sediment and randomly placed in radii from 0.1 to 4 m around the dispersal bags to promote *C. barbata* settlement. We used stones adjacent to our study areas, and not from the same area, to avoid disturbing the study site when cleaning the stones from organisms and sediment. The stones where cleaned to provide free substrate and avoid competition at the first stages of development of new recruits. After 4 days, the dispersal bags were removed from both restored sites.

*Ex situ* recruitment consisted of acquiring a supply of zygotes and culturing settlers in the laboratory. Fertile apical branchlets (around 2–3 cm in length) from the donor populations (March 2011, Fornells Bay) were collected and placed in plastic bags without seawater and transported to the laboratory under cold and dark conditions. Once in the laboratory, the bags containing the fertile branchlets were stored in the fridge (at 4°C and in dark conditions) for 12 h to promote zygote liberation. Concurrently, 16 natural flat schist stones with similar surface areas (approximately 0.04 m^2^) were placed at the bottom of ten 12-L tanks filled with filtered seawater, and fertile apical branchlets of *C. barbata* were placed on dispersal bags floating on the water surface of each tank for 4 days (Figures 2A,C). Moreover, some glass slides were placed on top of and between the stones to enable microscopically monitoring of zygote development during the first months (Figures 2F–J). zygote development to be microscopically monitored during the first month. For the first 4 days, the hydrodynamic conditions of the tank were kept as stable as possible to facilitate zygote settlement. Afterward, zygotes were cultured in a closed-water circuit with a renovation rate of 2 L per day using natural seawater at 21°C and natural light conditions. Seawater temperature was controlled with refrigerators (Hailea Chiller HC 500 A of Hailea). After 3 months (June 2011), stones with *C. barbata* recruits were transported to the restoration sites and six stones were placed at a distance of 25 m from the *in situ* restored area at each site (Figure 2E). It was not necessary to fix the stones since the restoration areas were extremely sheltered and the stones were heavy enough to prevent any movement.

### Monitoring the Restored and Reference Populations

After installing the *in situ* and *ex situ* recruitment set ups, both sites were visited monthly to ensure that the experiment was properly maintained. After five months, both *in situ* and *ex situ* recruits were large enough to allow visual density and height measurements. Then, the density (the total number of individuals per 0.04 m^2^) and the size structure distribution (the length of the main axis) of *C. barbata* individuals from each stone (approximately 20 × 20 cm) were monitored *in situ* twice in 2011 (August and November) and once during 2012, 2013, 2014, 2016, and 2017 (August) at each restored site and for each restoration technique.

At the beginning of the experiment, 3 natural *C. barbata* populations (Fornells Bay; Figure 1) were also selected as reference populations for comparison with the restored populations. The densities and size structure distributions of each reference population were monitored in 20 randomly distributed, 20 × 20-cm quadrats at the beginning and end of the experiment (i.e., August 2011, 2016, and 2017).

### Dispersal Capacity of the *in situ* Recruitment Method

At the same time, a new experiment was set up to explore the extension range of the *in situ* recruitment method. We studied the dispersion capacity of the *C. barbata* zygotes. For this purpose, we fixed a new pick (with 2 dispersal bags each) at each site, and six stones (approximately 0.04 m^2^ each) were placed just below the dispersal bags (0 m) along with six at a distance of 2 m, and finally six at a distance of 4 m. The dispersal bags were removed after 4 days, and the number of recruits from each stone was counted in August 2011 (Figure 2D).

### Data Analysis

#### Comparison of Techniques

To compare the two restoration techniques, the mean densities and size distribution at both restored sites were evaluated. The mean density (number of individuals/0.04 m^2^) over time was analyzed using a generalized linear mixed model (GLMM) with technique (2 levels: *ex situ* vs. *in situ*), site (2 levels: site 1 and site 2) and time (7 levels) as fixed factors, and stone as a random factor. Descriptive statistics were also calculated for the size structure distribution (the skewness and kurtosis) of restored populations and compared among both techniques and sites. The significance of the skewness and kurtosis values was calculated according to Sokal and Rohlf (1995).

#### Restoration Success

Restoration success was analyzed by comparing the final densities and size structures between restored and reference populations. The final density (August 2017) of restored populations was compared with that of reference populations by means of a generalized linear model (GLM) with one fixed factor with two levels (restored vs. control). Changes in the size structure distributions of the restored and reference populations over time were plotted using non-metric multidimensional scaling (MDS) to visualize their progression. The relative percentage of individuals in each size class (in 1-cm intervals) was the variable in the data matrix, and the Bray-Curtis distance (Bray and Curtis, 1957) with a dummy variable (= 1) was used to construct the similarity matrix.

#### Dispersal Capacity

Finally, the range in dispersal capacity obtained with the *in situ* method was analyzed using GLM, with site (2 levels) and distance from the dispersal bag (3 levels) as fixed factors. Pair-wise comparisons were also performed between distances.

GLMs and GLMMs are suitable for this kind of data since GLMs can handle non-normal data (Bolker et al., 2009) and GLMMs combine the properties of GLMs and linear mixed models, which incorporate random effects and therefore can cope with repeated measures over time (Pinheiro and Bates, 2000). All analyses were performed using the lme4 package (Bates et al., 2015) (Bates et al., 2015) for R software (R Core Team, 2016) and the statistical software Primer & Permanova v.6 (Clarke and Gorley, 2006).

### Costs

We compared the cost of restoring a population (25 m^2^) using the *ex situ* and *in situ* methods, considering the travel, transportation, personnel and material expenses (similarly to Carney et al., 2005). We did not consider the long-term monitoring costs since these costs are equivalent for the two techniques.

## Results

### Comparison of Techniques

**FIGURE 3:**
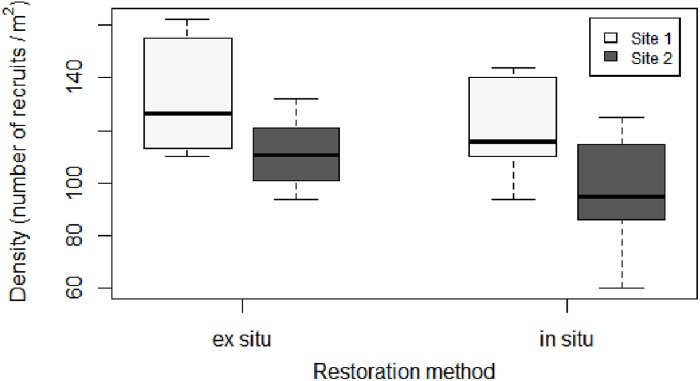
Boxplot of initial density (number of recruits/0.04 m^2^) for each restoration technique and site. In the boxplot, the bold horizontal line indicates the median value (Q2); the box marks the interquartile distances, Q1 and Q3; and the whiskers mark the values that are less than Q3+1.5^∗^IQR but greater than Q1–1.5^∗^IQR.

**FIGURE 4:**
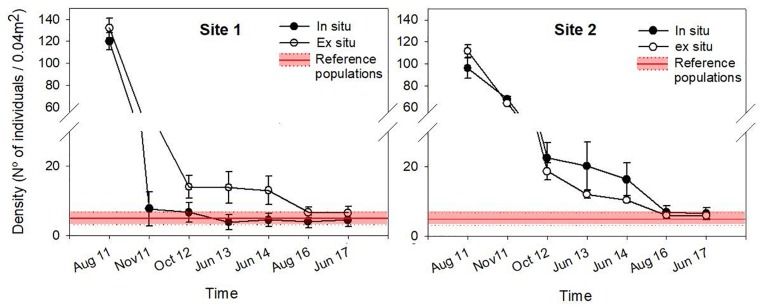
Mean density (± SE) through time for each restoration technique at each site. Reference population densities are represented in red, considering the mean and standard deviation values obtained from the reference populations in 2011, 2016, and 2017.

The density of recruits was similar between the two restoration techniques (Figures 3, 4 and Table 1). The mean initial densities ranged between 120 ± 7 recruits/0.04 m^2^ (site 1) and 96 ± 9 recruits/0.04 m^2^ (site 2) in the *in situ* experiment and between 132 ± 2 recruits/0.04 m^2^ (site 1) and 111 ± 9 recruits/0.04 m^2^ (site 2) in the *ex situ* experiment (Figure 3). No recruits were observed outside of the free substrate (stones) with the *in situ* method. The densities of the two restored populations greatly decreased during the first year but remained more stable afterward (Figure 4).

**Table 1:** Results of GLMM comparing the density (number of individuals/0.04 m^2^) through time in relation to the restoration techniques (*in situ* vs. *ex situ*).

Factor	*df*	*F*-value	*p*-value
Technique	1	0.11	0.43
Site	1	2.67	0.17
Time	6	796.26	**< 0.0001**
Technique ^∗^ Site	1	2.94	0.66
Technique ^∗^ Time	6	0.48	**< 0.0001**
Site ^∗^ Time	6	42.14	**< 0.0001**
Site ^∗^ Technique ^∗^ Time	6	21.25	**< 0.0001**


*For each factor, we report the degrees of freedom and the F- and *p*-values. The significant values are highlighted in bold in the table.*

**FIGURE 5:**
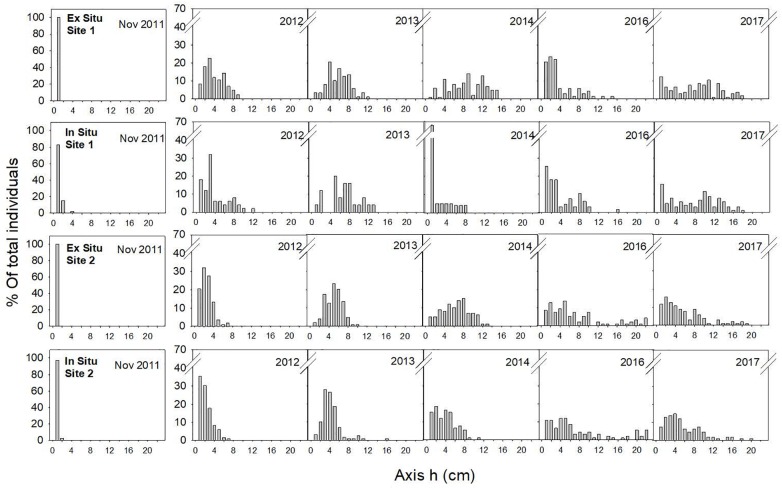
Size-class frequency distribution of the restored populations over time for each site and restoration technique. The X-axis represents the size-classes (length of the main axis) in 1-cm intervals, and the Y-axis represents the relative frequency of each size-class.

In November 2011, the main axes of almost all the individuals measured 1 cm, and one year later (August 2012), the skewness of the size-class structure was significantly positive, indicating the prevalence of small size-classes in the population. However, few individuals had reached axis lengths greater than 10 cm (Table 2 and Figure 5). Two years later (2013), all populations were approximately bell shaped and symmetric, with a large proportion of individuals having axis lengths between 2 and 5 cm, although some fertile individuals reached axis lengths of 14–16 cm (Table 2 and Figure 5).

**Table 2:** Characteristics of restored *C. barbata* populations through time and in relation to the restoration technique and site (N: number of *Cystoseira* individuals; h: length of the main axis (cm); g1: skewness; g2: kurtosis; Sig: significance of skewness and kurtosis values).

Date	Method	site	*N*	mean h	max h	g_1_	SE g_1_	sig. g_1_	g_2_	SE g_2_	sig. g_2_
2011 Aug	*in situ*	1	720	0,5	0,5	–	–	–	–	–	–
		2	576	0,5	0,5	–	–	–	–	–	–
	*ex situ*	1	793	0,5	0,5	–	–	–	–	–	–
		2	669	0,5	0,5	–	–	–	–	–	–
2011 Nov	*in situ*	1	46	0,83	4	3,25	0,35	**9,28**	13,59	0,69	**19,76**
		2	406	0,6	1,5	2,25	0,12	**18,58**	4,44	0,24	**18,37**
	*ex situ*	1	214	0,5	0,5	–	–	–	–	–	–
		2	384	0,5	0,5	–	–	–	–	–	–
2012	*in situ*	1	40	3,73	12	1,09	0,37	**2,92**	0,54	0,73	0,74
		2	135	1,99	6,5	1,15	0,21	**5,51**	0,81	0,41	1,96
	*ex situ*	1	84	3,89	8,5	0,4	0,26	1,52	-0,78	0,52	-1,50
		2	112	2,34	6,5	0,96	0,23	**4,20**	1,42	0,45	**3,13**
2013	*in situ*	1	26	6,81	13	-0,09	0,46	-0,20	-0,31	0,89	-0,35
		2	128	3,95	10,5	1,46	0,21	**6,82**	3,5	0,42	**8,24**
	*ex situ*	1	88	5,68	12	0,19	0,26	0,74	-0,09	0,51	-0,18
		2	103	4,88	10	0,1	0,24	0,42	-0,01	0,47	-0,02
2014	*in situ*	1	22	1,98	8	1,49	0,49	**3,03**	0,98	0,95	1,03
		2	91	3,75	11	0,63	0,25	**2,49**	0,1	0,50	0,20
	*ex situ*	1	85	8,55	15	-0,15	0,26	-0,57	-0,93	0,52	-1,80
		2	81	6,27	13	-0,05	0,27	-0,19	-0,58	0,53	-1,10
2016	*in situ*	1	67	3,94	16	1,25	0,29	**4,27**	1,67	0,58	**2,89**
		2	92	7,72	22	1,09	0,25	**4,34**	-0,05	0,50	-0,10
	*ex situ*	1	68	3,92	15	1,47	0,29	**5,06**	1,62	0,57	**2,82**
		2	94	7,52	22	1,13	0,25	**4,54**	0,21	0,49	0,43
2017	*in situ*	1	103	7,7	17,5	0,007	0,24	0,03	-1,17	0,47	**-2,48**
		2	110	5,54	20	1,29	0,23	**5,60**	1,68	0,46	**3,68**
	*ex situ*	1	105	8,12	18	0,11	0,24	0,47	-1	0,47	**-2,14**
		2	103	5,72	19	1,24	0,24	**5,21**	1,04	0,47	**2,21**


*These parameters are considered significant if the absolute value of the coefficient/standard error (SE) is greater than 2; the significant values are highlighted in bold in the table.*

In 2014, the size-class structures of the populations were symmetric and bell shaped, and most individuals were of intermediate size (Table 2 and Figure 5). One exception to this result was the population restored using the *in situ* method at site 1, where we found high mortality of large individuals but also high recruitment (Table 2 and Figure 5). These recruits were the result of new settlement events resulting from the already fertile restored individuals from 2013.

### Restoration Success

**FIGURE 6:**
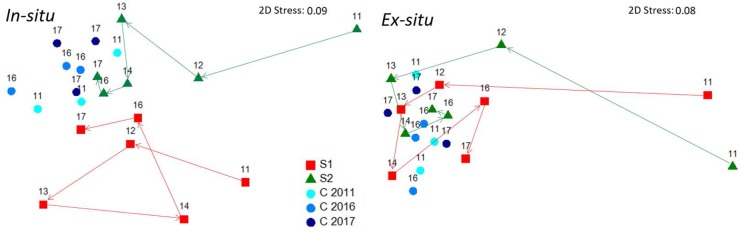
MDS ordination plot of the path followed by restored and natural populations over time for both *in situ* and *ex situ* techniques, according to the size-class data of each population. Numbers depicted over each point are years.

In 2017, six years after the restoration action, the size of each of the four restored *C. barbata* patches was roughly 25 m^2^. When comparing the final densities of restored populations with the densities of the reference populations (August 2017), no significant differences were observed (*F* = 0.08, *P* = 0.49; Figure 4). The evolution of the size-class distribution through time resulting from both techniques, sites and reference populations is illustrated in the MDS (Figure 6). The reference populations are displayed on the left side of the MDS (from 2011 to 2017), while the restored populations progressed from the right side in 2011 to the left side, ultimately moving closer to the reference populations. In 2014, the *in situ* restored population from site 1 returned to the right side of the MDS due to the mortality of large individuals and the high recruitment that was experienced (Figure 6). In 2016, all populations were located close to the reference populations, and they remained stable in 2017 (Figure 6).

### Dispersal Capacity

**FIGURE 7:**
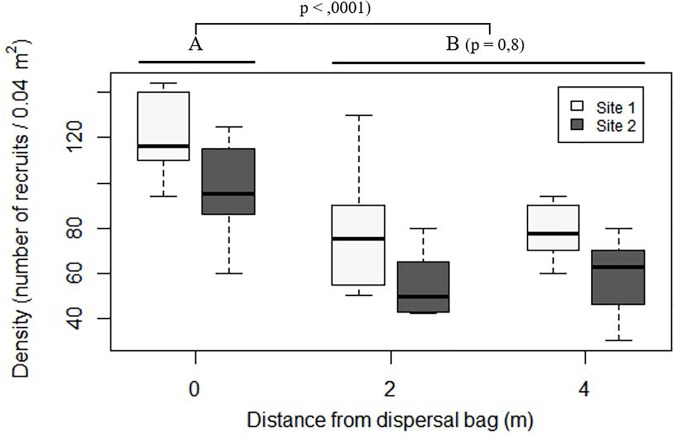
Boxplot of number of recruits on the stones placed *in situ* at increasing distances from the dispersal bags 5 months after their deployment. In the boxplot, the bold horizontal line indicates the median value (Q2); the box marks the interquartile distances, Q1 and Q3; and the whiskers mark the values that less than Q3+1.5^∗^IQR but greater than Q1–1.5^∗^IQR.

At both sites, stones situated below the dispersal bags (distance of 0 m) showed higher densities of *C. barbata* recruits than did those situated at distances of 2 and 4 m (*P* < 0.0001; Figure 7), while no differences were found between 2 and 4 m (*P* = 0.8; Figure 7).

### Costs

The cost of restoring 25 m^2^ of *C. barbata* forest ranged between 1,092 € using the *in situ* seeding technique and 2,665 € using the *ex situ* seeding technique (Table 3). The higher cost ascribed to the *ex situ* technique is related to the required infrastructure and the greater number of hours needed for culture maintenance.

**Table 3:** Cost for the different concepts required to restore an area of 25 m^2^ depending on the restoration technique used.

Concept	Rate	Cost	Total (€)
***Ex situ***			
Field time			
Collection	1h/2pax	40€/h^∗^pax	80
Ex-plant	3h/2pax	40€/h^∗^pax	240
Transport			
Car	200 km	0.40 €/km	80
			
Lab time			
Set up culture	4 h/2pax	40€/h^∗^pax	320
Culture maintenance	3 h/week^∗^pax	40€/h^∗^pax	1440
Materials			
Tanks	10	25 €	250
Water pump	1	60 €	60
Silicon Tubes	5 m	2 €/m	10
Epoxy	2	70 €/kg	140
Aerator	3	15 €	45
**TOTAL**			2665
***In situ***			
Field time			
Collection	1h/2pax	40€/h^∗^pax	80
Set up dispersal bags	4h/2pax	40€/h^∗^pax	320
Set up free substrate	3h/2pax	40€/h^∗^pax	240
Removal dispersal bags	1h/2pax	40€/h^∗^pax	80
Materials			
Iron Stick	16	7€/Pick	112
Epoxy	2	70 €/kg	140
Transport			
Car	300 km	0.40 €/km	120
**TOTAL**			1092


## Discussion

The present study is the first example of active restoration for locally extinct populations of habitat-forming fucoids using recruitment enhancement without adult transplantation of threatened populations, and these restored populations became self-sustaining, with densities and size-class structures comparable to those of the reference populations within five years. Active transplantation of adults or juveniles has been used as a mechanism to successfully restore habitat-forming species of fucoids (Susini et al., 2007; Campbell et al., 2014). The concept of recruitment enhancement has recently gained recognition as it applies to the restoration of threatened species (Yatsuya, 2010; Gianni et al., 2013; Falace et al., 2018). However, there have been only a few attempts at using this method, and most have been limited to the recruit stage with less than 1 year of monitoring (Stekoll and Deysher, 1996; Choi et al., 2000; Terawaki et al., 2003; Yu et al., 2012).

Here, we used recruitment enhancement methods to successfully restore a locally extinct *C. barbata* population with only one restoration action in 2011. Because the locally extinct population was unable to recover naturally, even thirty years after the primary stress had been ameliorated (Hoyo, 1981; Sales et al., 2011), we used seeding to overcome the limited natural dispersal rates that are typical of zygotes of the genus *Cystoseira* (Mangialajo et al., 2012), and we overrode the limited natural recruitment (Vadas et al., 1992; Capdevila et al., 2015) by cleaning the stones from organisms and sediment, providing free substrate to avoid competition. After six years, the sizes of each restored population was approximately 25 m^2^, which is comparable to the size-patches of natural *C. barbata* populations in Fornells Bay.

Recruitment was high and similar under both techniques, although a large proportion of recruits died during the first year. This sharp drop in density is common in natural populations due to the high natural sensitivity of the first fucoid life stages (Vadas et al., 1992; Irving et al., 2009). Although the density of individuals was similar between restored and reference sites in the second year following the restoration action, it took five years for the individuals of the restored populations to achieve comparable size-class structures to the reference ones. Thus, density is useful for monitoring success during the first period after a restoration action (recruits of settlers; here, 2 years), but after this stage, density should be complemented with other attributes, such as size structure, that will better describe the mature stage of the population.

Obtaining a *Cystoseira* population that reaches a well-represented and stable size distribution is the first goal for complete forest restoration. As for other structural species, the restoration success criteria should be linked to the recovery of the ecosystem function and services, and obtaining mature individuals that are able to self-sustain the new population is likely the first step for enhancing biodiversity and ecological processes. Complementary studies on the evolution of the associated community will probably elucidate whether the proposed indicators for population success may also be indicative of the overall recovery of ecosystem functions and services.

Both of the *in situ* and *ex situ* recruitment enhancement techniques applied here are probably suitable for other macroalgal species that produce large and fast-sinking zygotes with limited dispersion and that are poor competitors for space in their early stages (i.e., late-successional species). Thus, the techniques tested here could be used to restore other Mediterranean populations of *Cystoseira* spp., especially since the Council of Europe, specifically the Marine Strategy Framework Directive (United Nations Environment Programme/Mediterranean Action Plan [UNEP/MAP], 2013), pushes for active restoration to achieve a Good Environmental Status for a considerable number of habitats.

Knowledge of the biological traits of the target species will determine the choice between *in situ* and *ex situ* techniques. The *in situ* technique is especially recommended for species with high dispersal capacity, such as kelps, with a dispersal potential of hundreds of meters (Reed et al., 1988; Fredriksen et al., 1995). In contrast, the *ex situ* technique is more appropriate for species with a low dispersal capacity, such as *C. amentacea*, whose zygotes are not able to disperse a distance of even 40 cm (Mangialajo et al., 2012). Another benefit of the *ex situ* technique is that it minimizes the high mortality rates experienced by recruits and juveniles as a result of disturbances, predation or competition (Benedetti-Cecchi and Cinelli, 1992; Capdevila et al., 2015). The more the culture is prolonged, the more likely the critical life stages will be left behind, which ultimately enhances success. In our case, however, sources of mortality seemed to be rather irrelevant since the *ex situ* and the *in situ* survival rates were very similar during the first year. The *ex situ* technique should reduce the unpredictability of natural events and maximize success, while the *in situ* technique requires less infrastructure and maintenance, making it a cheaper option.

In summary, we provide a promising cost-effective method (consisting of two different techniques) that can be used to address the increasing need for the restoration of threatened species, especially fucoid forests. Moreover, we show that individual density is not a valid metric to assess the state of population recovery, and we propose the size distribution of the restored individuals as a suitable indicator of population maturity.

## Author Contributions

EC and MS conceived the ideas, designed the methodology, and established the restoration action. All authors were involved in collecting data during the monitored period. JV, MS, and EC wrote the manuscript, and all authors contributed critically to the drafts and gave their final approval for publication.

## Conflict of Interest Statement

The authors declare that the research was conducted in the absence of any commercial or financial relationships that could be construed as a potential conflict of interest.
